# High life history diversity within a single genus of algal viruses

**DOI:** 10.1093/ismejo/wraf146

**Published:** 2025-07-08

**Authors:** Eva J P Lievens, Irina V Agarkova, David D Dunigan, James L Van Etten, Lutz Becks

**Affiliations:** Aquatic Ecology and Evolution Group, Department of Biology, University of Konstanz, Konstanz, Germany; Department of Plant Pathology, University of Nebraska-Lincoln, Lincoln, Nebraska 68583-0722, United States; Nebraska Center for Virology, University of Nebraska-Lincoln, Lincoln, Nebraska 68583-0900, United States; Department of Plant Pathology, University of Nebraska-Lincoln, Lincoln, Nebraska 68583-0722, United States; Nebraska Center for Virology, University of Nebraska-Lincoln, Lincoln, Nebraska 68583-0900, United States; Department of Plant Pathology, University of Nebraska-Lincoln, Lincoln, Nebraska 68583-0722, United States; Nebraska Center for Virology, University of Nebraska-Lincoln, Lincoln, Nebraska 68583-0900, United States; Aquatic Ecology and Evolution Group, Department of Biology, University of Konstanz, Konstanz, Germany

**Keywords:** aquatic virus, phycodnavirus, *chlorovirus*, *Chlorella*, functional traits, viral growth rate, viral survival, life cycle

## Abstract

Microbial viruses are key players in aquatic ecosystems, where they control host populations and affect nutrient flow. The impact of these viruses can be understood through their life history traits, which are used to parameterize ecological models and infer evolutionary strategies. However, most existing data on microbial virus traits come from highly divergent strains. Very little is known about the trait diversity of closely related viruses, opening the critical question: can unknown viral traits be extrapolated from those of known strains? To answer this question, we quantified the life history diversity of related aquatic microbial viruses in unprecedented detail. We measured nine life history traits in 34 strains belonging to the phytoplankton-infecting genus *Chlorovirus*. Chloroviral traits varied 5- to 77-fold across strains, in some cases rivaling the known trait range for all phytoplankton viruses. Contrary to expectations, only specific infectivity was predictive of viral growth and there was no evidence of life history trade-offs. Our results suggest that more detailed studies of viral diversity could change our understanding of their function in aquatic ecosystems. More broadly, we show that known virus strains may not be representative of their relatives.

The role of microbial viruses in aquatic ecosystems can be understood by studying the traits that determine viral fitness: their life history or “performance” traits [1]. For lytic viruses, commonly measured life history traits include adsorption rate, lysis time, burst size, and mortality rate. These traits can be combined to model viral growth, and thus to estimate viral impact on host populations and ecosystems [2]. Life history traits are also used to look for overarching constraints and patterns in viral evolution [3–6]. Aquatic microbial viruses have highly diverse life histories [5–7], but very little is known about their diversity at the genus and species level. Logistical and sampling constraints mean that studies comparing >3 related viruses are rare [8–10] and often limited to 1–2 traits [8, 9]. This makes it hard to judge whether known viruses are representative of their relatives and limits our ability to make predictions about diverse aquatic viral communities.

We used the genus *Chlorovirus* (family *Phycodnaviridae*) to illuminate the life history diversity of related viruses. The chloroviruses are lytic dsDNA viruses that infect freshwater phytoplankton [11]; their life cycle [12] and phylogeny [13, 14] are exceptionally well-characterized. We quantified detailed life histories of 34 chlorovirus strains belonging to subgenera Alpha*-* and Gammachlorovirus (Supplementary Table S1). We then compared the *Chlorovirus* trait diversity with that of other phytoplankton viruses and explored its ecological and evolutionary impact.

We quantified the chlorovirus’ life history as follows (Fig. 1A and Supplementary Table S2) [12]. Chloroviruses first adsorb to the host at a rate determined by the adsorption constant *k*. They then digest a hole in the cell wall, fuse their internal membrane with the host’s plasma membrane, and depolarize the plasma membrane with depolarization probability *d*. Depolarization is followed by viral genome entry, replication, and release of progeny virions (virus particles) through lysis. The probability that these steps are successful is the release probability *r*. Release timing is described by the mean ± SD of lysis time *μ_l_* ± *σ_l_*; the number of progeny virions is the burst size per depolarized cell *b_d_* or per release *b_r_*. The overall probability that a virion can attach, depolarize, and release progeny (i.e. that a virion is infectious) is the specific infectivity *s*. In the absence of available hosts most infectious virions decay following mortality rate *m*, but a persistent fraction *p* resists decay. All traits were measured using modified one-step growth and survival assays, which generate an estimate and 95% CI for every trait ([12], Supplementary Methods). Viruses were assayed in their type hosts (Fig. 1, Supplementary Table S1); assays were run simultaneously to minimize environmental variation.

**Figure 1:**
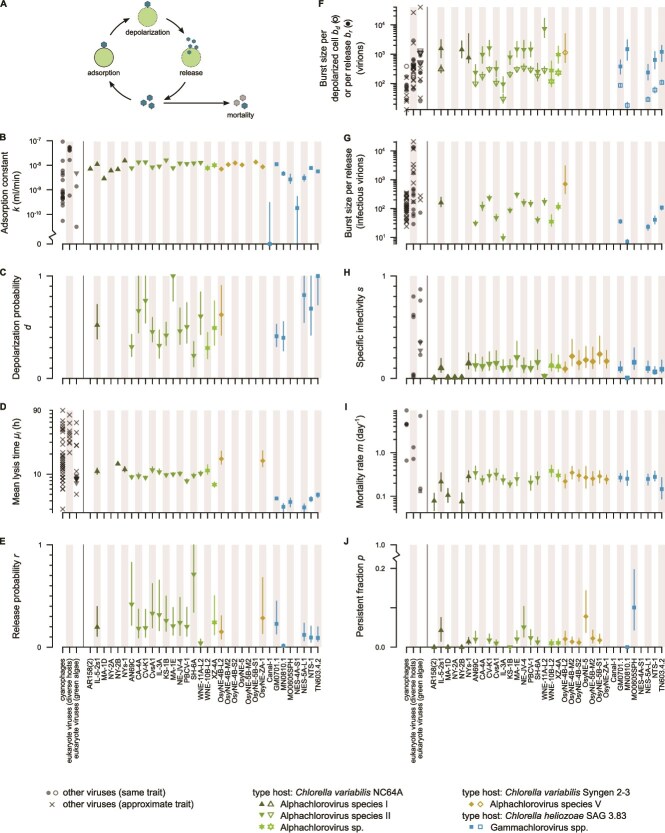
Life history of the chloroviruses. (A) Overview of the chlorovirus life cycle. (B–J) Life history traits. Chlorovirus data are shown as point estimates with 95% CIs; blanks indicate poorly resolved estimates that were excluded from further analysis (Supplementary Table S3). Chlorovirus data were compared to published values for phytoplankton viruses in permissive environments (dark gray) following [5, 6]. Published values were “approximate” if we manually inferred the mean lysis time, or if it was unclear whether burst size was measured per lysed or infected (≈depolarized) cell. SD of lysis time was tightly correlated with mean lysis time and is not shown. See Supplementary File 2 for details and references.

The chlorovirus traits were highly diverse, with trait estimates varying 5- to 77-fold between strains (Fig. 1B–J). Some of this diversity was explained by the type host (49%–72% of variance for lysis time and burst size per depolarized cell, *P* < .01; 1%–28% for other traits, *P* ≥ .06; Supplementary Table S4), which may be an effect of host differences or viral phylogeny (confounded factors, see Fig. 1 legend). *Chlorovirus* burst sizes spanned most of the known trait range for phytoplankton viruses (Fig. 1F and G). This is particularly striking because the known trait range represents a wide range of viral genome types, morphologies, host genera, and abiotic environments [5–7], whereas the chlorovirus trait range does not. In contrast, *Chlorovirus* lysis time, specific infectivity, and mortality rate had comparatively restricted trait ranges (Fig. 1D, H, and I). This restriction might be a proximate consequence of shared host physiology (e.g. fast host growth causing fast lysis [5]) or an ultimate consequence of shared ecological conditions (e.g. unpredictable host availability selecting for slow mortality).

To explore how trait variation affects population dynamics, we predicted viral growth rates from our estimates of specific infectivity, adsorption constant, mean lysis time, and burst size per release ([3], Supplementary Methods). The predicted growth rates correlated with observed growth rates in lab assays (*ρ* = 0.51, *P* = .02; Fig. 2A), indicating that trait variation was relevant for population dynamics. However, not all traits were equally relevant: regression models showed that only specific infectivity was predictive of observed growth rates (present in all models with ΔAIC ≤ 2, *n* = 21 strains; Fig. 2A). Future work should test whether trait relevance is context-dependent, for example whether adsorption is more important when host densities are lower.

**Figure 2:**
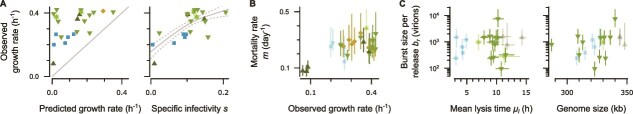
Viral growth and potential trade-offs. (A) Trait basis of viral growth. Left: Observed growth rates were compared with growth rates predicted from the adsorption constant, mean lysis time, specific infectivity, and burst size (gray: 1:1 line). Right: Of the aforementioned traits, only specific infectivity had a detectable effect (gray: prediction ± SE of the relevant regression model). (B and C) Trait correlations. Correlations were only tested within species where *n* ≥ 5 (growth vs. mortality in Alphachlorovirus species I, species II, species V; burst size vs. lysis time in Alphachlorovirus species II; burst size vs. genome size in Alphachlorovirus species II). Trait estimates for species where correlations were not tested are shown as translucent. Correlation analyses were weighted by 95% CI breadth. Symbols and colors as in Fig. 1.

To examine whether life history trade-offs constrain chlorovirus evolution, we looked for correlations among trait estimates. We tested correlations consistent with three trade-offs that are hypothesized to affect lytic viruses: growth rate versus mortality rate [3, 4], burst size versus lysis time [4], and burst size versus genome size [6]. Correlations were tested within species where *n* ≥ 5 (Fig. 2; Supplementary Methods). None of the hypothesized trade-offs were supported (*P* ≥ .47; Fig. 2B and C). These results are inconclusive: it is possible that the trade-offs do not apply to the chloroviruses (e.g. because they are mitigated by other functions in the large chlorovirus genome [6]) or that they were obscured by other differences between the strains [15]. Experimental evolution could be used to distinguish between the two possibilities [4, 16].

In summary, we found that the *Chlorovirus* genus contains high diversity in traits with direct effects on fitness. The scale of *Chlorovirus* trait diversity is remarkable when compared to that of other phytoplankton viruses, particularly for burst size (Fig. 1F–G). We assume that the chloroviruses are not unique, and that other genera of microbial viruses can be similarly diverse. If so, extrapolating life history traits from known viruses could lead to errors when predicting the dynamics of a diverse virus population, and complicate the inference of viral life history strategies (e.g. burst size versus virion size [6]). Exploring the phenotypic diversity of microbial viruses at the genus and species level is therefore an essential next step to understanding their function in aquatic ecosystems. More broadly, our results imply that the life histories of known viruses may not be representative of their species or genus, and argue for more attention to diversity across viral ecology.

## Supplementary Material

Supplementary_material_wraf146_File_1

Supplementary_material_wraf146_File_2

## Data Availability

The data and code underlying this article are available in Zenodo at https://doi.org/10.5281/zenodo.6573769 and https://doi.org/10.5281/zenodo.13999011.

## Supplementary material

Supplementary File 1 contains the Supplementary Methods and Supplementary Tables.
