# Supplementary material for: High life history diversity within a single genus of algal viruses
Source: ISME J. 2025 Jul 8;19(1):wraf146. doi: 10.1093/ismejo/wraf146 (PMC12319314; doi:10.1093/ismejo/wraf146)
Supplement: Supplementary_material_wraf146_File_1 [file supplementary_material_wraf146_file_1.pdf]

Supplementary Tables

Table S1. List of viral strains. All isolates/strains can be found at NCBI_BioProject PRJNA1154233. Strains were collected from freshwater environments in Australia (AN69C), Argentina (AR158), Japan (CV-K1 and CvsA1), Guatemala (GM0701.1), China (XZ-4A), an unknown location (SH-6A), and the USA (all other strains). Species assignments following Carvalho et al. [1] and Henriques et al. [2].

Subgenus	Species	Strains	Type host
Alphachlorovirus	species I	AR158(2)*, IL-5-2s1, MA-1D, NY-2A, NY-2B, NYs-1	NC64A
	species II	AN69C, CA-4A, CV-K1 (also called CviKI), CvsA1, IL-3A, KS-1B, MA-1E, NE-JV-4, PBCV-1, SH-6A, WNE-11A-L2 (also called WNE-11A-L1)	NC64A
	species V	OSyNE-4B-L2, OSyNE-4B-M2, OSyNE-4B-S2, OSyNE-5, OSyNE-5B-M2, OSyNE-5B-S1, OSyNE-ZA-1	Syngen 2-3
	unknown	WNE-10B-L2, XZ-4A	NC64A
Gammachlorovirus	species I	TN603.4.2	SAG 3.83
	species II	GM0701.1	SAG 3.83
	species III	MO0605SPH	SAG 3.83
	species IV	NTS-1	SAG 3.83
	species VII	NES-5A-L1	SAG 3.83
	species VIII	Canal-1, NES-4A-S1	SAG 3.83
	species X	MN0810.1	SAG 3.83

*Suffix (2) used to distinguish this lysate from another AR158 lysate in our collection.

Table S2. Traits estimated in the mOSG and mS assays.

Life cycle phase	Symbol	Trait	Definition	Assay
Reproduction	k	adsorption constant	constant that determines the rate at which virions adsorb to host cells	mOSG
	d	depolarization probability	probability that an adsorbed virion is capable of depolarizing a host cell (can be quantified through the variation in initial virion:host ratio)	mOSG
	μ_l	mean lysis time	mean time until lysis (assuming all release events produce the same number of virions)	mOSG
	σ_l	standard deviation of lysis time	standard deviation of time until lysis (assuming all release events produce the same number of virions)	mOSG
	r	release probability	probability that a depolarized cell releases infectious virions	mOSG & mS
	b_d	burst size per depolarized cell	average number of virions produced by a depolarized cell	mOSG
	b_r	burst size per release	average number of virions produced per release event	mOSG & mS
	s	specific infectivity	initial proportion of infectious virions (equivalent to the probability that a virion is capable of adsorbing, depolarizing, and releasing infectious progeny)	mS
Survival	m	mortality rate	rate at which nonpersistent infectious virions became noninfectious	mS
	p	persistent fraction	proportion of persistent infectious virions, i.e. of infectious virions that resisted decay for the duration of the assay (4 weeks)	mS

Table S3. Trait estimates excluded from further analysis.

Reason	Assay	Traits excluded	Strains
Incomplete assays	mOSG	k, d, μ_l, σ_l, b_d, r, b	OSyNE-5B-M2
		d, μ_l, σ_l, b_d, r, b_r	AR158(2), Canal-1, MA-1D, NES-4A-S1, NY-2A, OSyNE-4B-M2, OSyNE-4B-S2, OSyNE-5, OSyNE-5B-S1
	mS	s, m, p	Canal-1, NES-4A-S1
		m, p	NY-2A
Poor model fit	mOSG	d, b_d, r, b_r	MO0605SPH
Uninformative CIs	mOSG	d	NY-2B, NYs-1, OSyNE-ZA-1
		b_d	NY-2B, NYs-1, OSyNE-ZA-1
		r	NY-2B, NYs-1, WNE-10-L2
		b_r	OSyNE-4B-L2, OSyNE-ZA-1
	mS	m	MO0605SPH, NE-JV-4, WNE-11A-L2
		p	CA-4A, IL-3A, GM0701.1, NES-5A-L1, NTS-1, OSyNE-ZA-1, TN603.4.2, WNE-11A-L2

Table S4. Viral trait variance within and among type host groups. Type host groups: *C. variabilis* NC64A (Alphachlorovirus species I, species II, and sp.), *C. variabilis* Syngen 2-3 (Alphachlorovirus species V), *C. heliozoae* SAG 3.83 (Gammachlorovirus spp.). Groups were not included if $n < 3$. Abbreviations: SS, sum of squares; res., residuals. SS(groups)/SS(total) is the proportion of variance that can be attributed to differences among groups. Group effects that were significant after correction for multiple testing are underlined.

Symbol	Trait	groups tested	SS(groups)	SS(res.)	SS(groups) /SS(total)	F	P	corrected P
k	adsorption constant	all	133	1514	0.09	1.3	0.29	0.96
d	depolarization probability	NC64A, SAG 3.83	0.17	1.91	0.08	1.5	0.24	0.96
μ_l	mean lysis time	NC64A, SAG 3.83	1019	405	0.72	50.3	< 0.001	< 0.001
σ_l	standard deviation of lysis time	NC64A, SAG 3.83	25.4	18.2	0.58	27.9	< 0.001	< 0.001
r	release probability	NC64A, SAG 3.83	0.07	0.29	0.19	3.8	0.07	0.41
b_d	burst size per depolarized cell	NC64A, SAG 3.83	14.4	14.7	0.49	16.6	< 0.001	0.01
b_r	burst size per release	NC64A, SAG 3.83	0.13	13.3	0.01	0.2	0.67	1.00
s	specific infectivity	all	0.02	0.06	0.28	5.6	0.01	0.06
m	mortality rate	all	0.01	0.19	0.04	0.5	0.62	1.00
p	persistent fraction	NC64A, Syngen 2-3	249	2368	0.10	2.0	0.17	0.87

Note on the supplementary methods

In this study, we used recently-developed methods to characterize 34 chlorovirus strains. Four of these strains were also included in Lievens et al. [3]*, where they illustrated the development of the experimental and analytical methods. As some aspects of the analytical workflow have to be run on the entire dataset**, the raw data and kinetic models for all 34 strains were made available at https://doi.org/10.5281/zenodo.6573769 upon publication of Lievens et al. [3]. Curated data and code for the current analyses are available at https://doi.org/10.5281/zenodo.13999011 .

*Alphachlorovirus strains AN69C, CV-K1, KS-1B, and PBCV-1.

**Specifically the decision to simplify the kinetic models, see Lievens et al. [3] Figs. S8 and S10.

Supplementary methods

Viruses, algae, and experimental conditions

We phenotyped 34 chloroviruses belonging to the subgenera Alpha- and Gammachlorovirus (Table S1). Viruses belonging to Alphachlorovirus species I and species II infect the ciliate endosymbionts *Chlorella variabilis* NC64A and *C. variabilis* Syngen 2-3. Their type host is *C. variabilis* NC64A. Viruses belonging to Alphachlorovirus species V have diverse host ranges; we tested a set of strains that can only replicate in *C. variabilis* Syngen 2-3. Viruses belonging to the subgenus Gammachlorovirus infect the heliozoon endosymbiont *Chlorella heliozoae* SAG 3.83. All of the described procedures took place in the type hosts (Table S1).

The chlorovirus strains were isolated from natural ponds or streams around the world between 1981 and 2017 (Table S1), and have been maintained as part of a stock collection since then. Virus suspensions were stored in lysate form at 4°C. Before starting the assays, we refreshed and amplified the virus stocks: 0.5 ml of the lysate was inoculated into 10 ml of 2×10^6 algae/ml suspension of the type host, incubated until the algae lysed (24 h or 48 h), and filtered through 0.2 µm. The resulting filtrates were stored at 4°C and the concentration of virus particles (virions) was measured by flow cytometry [3, 4]. The filtrates with insufficient virion concentrations were given one more round of this amplification treatment.

The algal strains NC64A, Syngen 2-3, and SAG 3.83 were stored on agar slants at 4°C and inoculated into liquid medium before use. We used a modified version of Bold’s Basal Medium [BBM, 5], with ammonium chloride substituted for sodium nitrate as a nitrogen source and double the concentration of trace element solution 4 [first used by 6]. We keep the abbreviation BBM in order

to distinguish our medium from the enriched "MBBM" typically used in this model system [e.g. 7]. Algae used in the assays were in late exponential phase (~2×10^6 algae/ml in this medium).

Unless otherwise specified, assays took place under our typical growth conditions for Chlorella algae: 20°C and constant light. Algal growth and virus incubations were done on an orbital shaker with diameter 10 mm and frequency 120 rpm. Any 4°C storage was also dark storage.

Measurement of life history traits

We measured the chlorovirus' life history traits using modified one-step growth (mOSG) and modified survival (mS) assays, as described in Lievens et al. [3] and on protocols.io [8, 9]. Briefly:

- The mOSG assay tracked virion concentrations over the course of a single replication cycle. Algae and viruses were combined in small volumes of liquid culture (5 replicates) and mixed for 15 min to induce adsorption. Cultures were then synchronized by 1000-fold dilution and sampled every 2 h for 16 h. The free virion concentration in each sample was quantified by flow cytometry. A kinetic model was fit to the resulting one-step growth curves and used to estimate point estimates and 95% CIs for most of the traits shaping viral reproduction (Table S2).

  An important aspect of the mOSG assay is that replicate infections were initiated at varying virion:host ratios (0.5, 1, 2, 5, or 10), which enabled the quantification of depolarization probability. This is described as follows in Lievens et al. [3]: "as MOP [virion:host ratio] increases, the proportion of depolarized host cells approaches 1. Since depolarization prevents coinfection (21), we can assume that each depolarized host cell produces the same average number of progeny virions [...]. Therefore, the total number of progeny virions approaches a maximum as MOP increases, and the depolarization probability can be derived from the rate of this increase."
- The mS assay tracked the decline of infectious virions in the environment when exposed to 20°C and constant light. Freshly lysed viral suspensions were separated from their hosts and aliquoted into small volumes. After 0, 7, 14, 21, and 28 days, we used most probable number-like methods to quantify the concentration of infectious virions in each suspension. A biphasic decay model was fit to this data and used to estimate point estimates and 95% CIs for the specific infectivity and survival traits (Table S2).
- By comparing the mOSG and mS assays, we were able to estimate point estimates and 95% CIs for the two remaining reproduction traits (Table S2).

After fitting the kinetic models, we evaluated the fits and trait estimates. We excluded trait estimates from incomplete assays (d, μ_l, σ_l, b_d, r, and b_r for strains that did not finish growing in the

mOSG assay; *m* and *p* for strains that did not decay in the mS assay; all traits for assays that failed), estimates derived from poorly fitting models, and estimates with uninformatively broad CIs compared to the plausible phenotypic range (criteria: CI > 0.67 for *d*, *s*, and *r*; CI spanning more than an order of magnitude for b_d, *p*, and b_r; CI overlapping with the upper fitting constraint for *k*, μ_l, σ_l, and *m*). Details on exclusion are provided in Table S3.

A second evaluation step is to examine the effect of correlations between model parameters [compensating effects, 3]. These correlations are caused by random noise and are inherent to the model fitting. For example, if the initial concentration of infectious virions in the mS assay is estimated to be slightly higher/lower than the true value, then model fitting can lead to an over-/underestimate of both the specific infectivity and the mortality rate. Lievens et al. found that correlations occur between parameters *d* & b_d, μ_l & σ_l, μ_l & b_d, σ_l & b_d, *s* & *p*, and *s* & *m* [Fig. S4 in 3]. A visual assessment of these effects in the current dataset is presented in Fig. S1.

The biological assumptions underlying the mOSG and mS assays are discussed in detail in Lievens et al. [3]. Here we highlight two assumptions that are particularly relevant to the current dataset. First, we assumed that all virions were capable of adsorbing to host cells. If a non-negligible proportion of virions could not adsorb (in all or some strains), then we have underestimated the adsorption constant and release probability and overestimated the burst size per release (in all or some strains). Second, we assumed that the most probable number-like approach used in the mS assay accurately measured the infectiousness of virus suspensions. Specifically, we assumed that all algal cultures inoculated with ≥ 1 infectious virion lysed within 4 days. This may not be the case for slow-growing strains (e.g. MA-1D, NY-2B), which would lead to underestimates of the specific infectivity and release probability and overestimates of the burst size per release.

All analyses were performed in R version 4.2.2 [10].

Fig. S1 (next page). Effect of correlations between model parameters arising from the fitting process. To look for confounding effects of the correlations between parameters *d* & b_d, μ_l & σ_l, μ_l & b_d, σ_l & b_d, *s* & *p*, and *s* & *m*, we compared the bootstrapped CIs to the overall phenotype space. Each panel shows a parameter combination, and each polygon represents one viral strain. Colors as in Fig. 1: green shades indicate Alphachlorovirus species I, species II, and unknown (tested in *C. variabilis* NC64A), gold indicates Alphachlorovirus species V (tested in *C. variabilis* Syngen 2-3), and blue indicates Gammachlorovirus strains. Polygons outline the bootstrap values that fell within the 95 % CIs. Example interpretation 1: there is little overlap between polygons for μ_l and b_d, so parameter correlations do not need to be considered further. Example interpretation 2: the overlapping polygons for *s* and *p* indicate that correlations caused by model fitting cannot be distinguished from true phenotypic correlations.

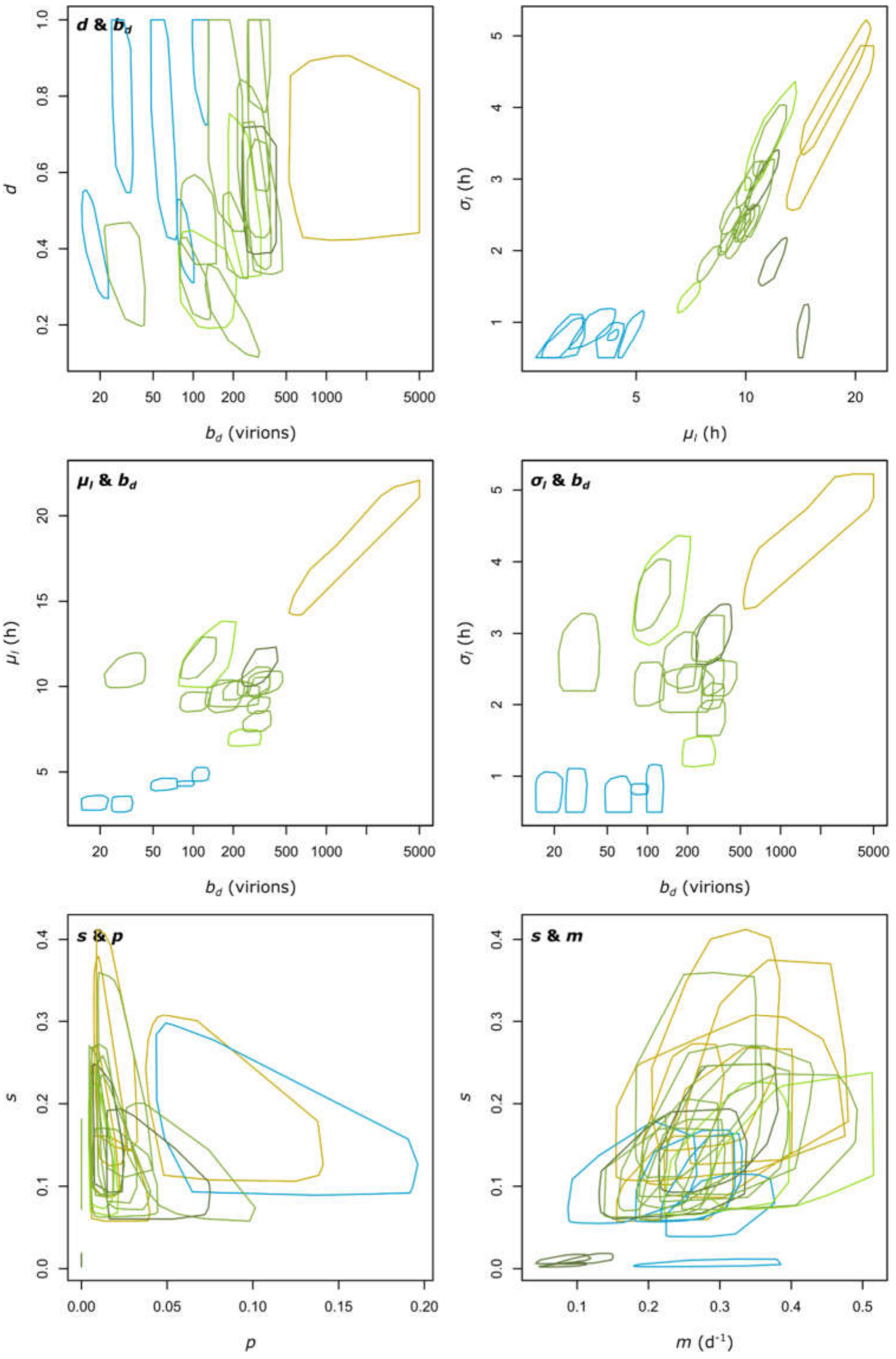

Analysis of trait diversity

We partitioned the *Chlorovirus* trait diversity across the three type hosts: *C. variabilis* NC64A, *C. variabilis* Syngen 2-3, and *C. heliozoae* SAG 3.83. Groups were not included if $n < 3$. ANOVAs were weighted by the inverse of the relative CI breadth, where the relative CI breadth is (CI breadth/point estimate). The burst size per depolarized cell, burst size per release, and persistent fraction were *ln*-transformed to normalize their distributions. Correction for multiple testing was done with Holm's

method [11]. It is important to note that partitioning trait diversity across type hosts cannot identify the underlying causal factors, which may be physiological differences between the host types or phylogenetic differences between the virus strains (which are confounded with type host, Fig. 1 legend, Table S1).

We also compared the *Chlorovirus* trait diversity with published data on other phytoplankton viruses. To obtain trait data, we combined data from published reviews with updates from a systematic literature search. For optimal comparability, we only retained phenotypes obtained under permissive conditions (growth traits, [following 12, 13]) or well-lit conditions (mortality rate). Where necessary, data was extracted manually using WebPlotDigitizer version 4 [14]. For the precise search procedure, search terms, extracted data, and references, see Supplementary File 2.

- For the adsorption constant, we used the data collected by Edwards et al. [13] as a basis and searched Web of Science for estimates published since 2020.
- For lysis time and burst size, we used the data collected by Edwards & Steward [12] as a basis and searched Web of Science for estimates published since 2018. For any papers that reported the earliest lysis time (latent period) or latest lysis time, we extracted the approximate mean lysis time (the half-way point of the virus rise curve or of the host lysis curve). We noted whether burst size was measured in virions or infectious virions. We also noted whether burst size was calculated per infected cell (most similar to our burst size per depolarized cell) or per lysed cell (most similar to our burst size per release); if this was unclear we scored the trait as approximate.
- For specific infectivity, we searched Web of Science for published estimates.
- For mortality rate, we used the data collected by Mojica & Brussaard [15] as a basis and searched Web of Science for estimates published since 2014.

Growth assay

We used the refreshed filtrates prepared at the start of the mS assay to measure viral growth. Viruses and algae were combined in a 96-well culture plate (2 wells per strain) to a final volume of 0.2 ml containing 1×10^6 algae/ml and 2,500 or 25,000 virions/ml (virion:host ratio 0.0025 or 0.025). The suspensions were incubated for 24 h. After 24 h, suspensions were centrifuged for 15 min at 2000 g to separate algae and viruses, and the virion concentration of the supernatants was measured by flow cytometry [3, 4]. We calculated the observed growth rate as *ln*(mean of the final/initial virion concentration for the 2 replicates)/24.

The predicted growth rate was calculated as De Paepe & Taddei's multiplication rate $s*b_r*\mu_l^{-1}$ [16] with an additional adsorption term: $(1 - e^{-k*A_g}) * s * b_r * \mu_l^{-1}$ or equivalently $(1 - e^{-k*A_g}) * d * b_d * \mu_l^{-1}$. Here A_g is the algal concentration during the growth assay, i.e. 1×10^6 algae/ml.

The observed and predicted growth rate were compared using Spearman's rank correlation. To examine whether all component traits contributed to the variation in observed growth rate, we regressed the observed growth rate onto the adsorption constant *k*, specific infectivity *s*, mean lysis time μ_l, and *ln*-transformed burst size per release b_r. We allowed the observed growth to be a nonlinear function of the trait values, but we forced the functions to be mechanistically sensible: adsorption, specific infectivity, and burst size were constrained to positive monotonic effects, while lysis time was constrained to a negative monotonic effect (shape-constrained additive models, package "scam" version 1.2-17, [17]). We compared models with all additive trait combinations using the Akaike information criterion [AIC, 18]; traits present in all models with ΔAIC ≤ 2 were considered to be predictive. Analyses were performed in R version 4.2.2 [10].

Signals of trade-offs

Finally, we looked for signals of three trade-offs that are commonly hypothesized to affect lytic viruses: growth rate vs. mortality rate [16, 19], burst size vs. lysis time [19], and burst size vs. genome size [13]. To avoid phylogenetic artifacts we tested within virus species; we did not test correlations if $n < 5$. Correlations were tested using Spearman's rank correlations, weighted by the precision of each estimate (1/√(product of the relative CI breadth of trait 1 and trait 2), package 'weights' [20], where the relative CI breath is (CI breadth/point estimate)). Holm's method was used to correct for multiple testing [11]. Analyses were performed in R version 4.2.2 [10].

References for Supplementary File 1

1. Carvalho JVRP et al. Genomics and evolutionary analysis of *Chlorella variabilis*-infecting viruses demarcate criteria for defining species of giant viruses. *J Virol* 2024;e0036124. https://doi.org/10.1128/jvi.00361-24
2. Henriques LR et al. Revealing the hidden diversity of *Chlorella heliozoae*-infecting giant viruses. *Npj Viruses* 2025. https://doi.org/10.1038/s44298-025-00088-y
3. Lievens EJP et al. Efficient assays to quantify the life history traits of algal viruses. *Appl Environ Microbiol* 2023;**89**:e01659-23. https://doi.org/10.1128/aem.01659-23
4. Lievens EJP. Virion quantification by flow cytometry, without fixation or freezing. *protocols.io*. dx.doi.org/10.17504/protocols.io.6qpvr6q93vmk/v1. (2022, date last accessed).
5. Nichols HW, Bold HC. *Trichosarcina polymorpha* Gen. et Sp. Nov. *J Phycol* 1965;**1**:34–38. https://doi.org/10.1111/j.1529-8817.1965.tb04552.x
6. Frickel J, Sieber M, Becks L. Eco-evolutionary dynamics in a co-evolving host-virus system. *Ecol Lett* 2016;**19**:450–459. https://doi.org/10.1111/ele.12580
7. Van Etten JL et al. Growth cycle of a virus, PBCV-1, that infects *Chlorella*-like algae. *Virology* 1983;**126**:117–125. https://doi.org/10.1016/0042-6822(83)90466-X
8. Lievens EJP. Modified one-step growth (mOSG) assay. *protocols.io*. dx.doi.org/10.17504/protocols.io.4r3l2op9pv1y/v3. (2023, date last accessed).
9. Lievens EJP. Modified survival (mS) assay. *protocols.io*. dx.doi.org/10.17504/protocols.io.81wgb6knylpk/v3. (2023, date last accessed).
10. R Core Team. R: A language and environment for statistical computing. 2020. Vienna, Austria: R Foundation for Statistical Computing, 2020.
11. Holm S. A simple sequentially rejective multiple test procedure. *Scand J Stat* 1979;**6**:65–70. https://doi.org/10.2307/4615733
12. Edwards KF, Steward GF. Host Traits Drive Viral Life Histories across Phytoplankton Viruses. *Am Nat* 2018;**191**:566–581. https://doi.org/10.1086/696849
13. Edwards KF, Steward GF, Schvarcz CR. Making sense of virus size and the tradeoffs shaping viral fitness. *Ecol Lett* 2021;**24**:363–373. https://doi.org/10.1111/ele.13630
14. Rohatgi A. WebPlotDigitizer: Version 4.8. https://apps.automeris.io/wpd4/ (2024, date last accessed)
15. Mojica KDA, Brussaard CPD. Factors affecting virus dynamics and microbial host–virus interactions in marine environments. *FEMS Microbiol Ecol* 2014;**89**:495–515. https://doi.org/10.1111/1574-6941.12343
16. De Paepe M, Taddei F. Viruses' life history: towards a mechanistic basis of a trade-off between survival and reproduction among phages. *PLOS Biol* 2006;**4**:e193. https://doi.org/10.1371/journal.pbio.0040193
17. Pya N. scam: Shape Constrained Additive Models. 2024.
18. Akaike H. A new look at the statistical model identification. *IEEE Trans Autom Control* 1974;**19**:716–723. https://doi.org/10.1109/TAC.1974.1100705
19. Goldhill DH, Turner PE. The evolution of life history trade-offs in viruses. *Curr Opin Virol* 2014;**8**:79–84. https://doi.org/10.1016/j.coviro.2014.07.005
20. Pasek J. weights: Weighting and Weighted Statistics, version 1.0.4. 2021.
